# Supplementary material for: Prevalence and determinants of using complementary and alternative medicine for the treatment of chronic illnesses: A multicenter study in Bangladesh
Source: PLoS One. 2022 Jan 5;17(1):e0262221. doi: 10.1371/journal.pone.0262221 (PMC8730415; doi:10.1371/journal.pone.0262221)
Supplement: S1 Fig — (DOCX) [file pone.0262221.s001.docx]

# S1: Types of CAM utilised by the patients
