# Supplementary figures and images for: Prevalence and determinants of using complementary and alternative medicine for the treatment of chronic illnesses: A multicenter study in Bangladesh
Source: PLoS One. 2022 Jan 5;17(1):e0262221. doi: 10.1371/journal.pone.0262221 (PMC8730415; doi:10.1371/journal.pone.0262221)

# S2: Relationship between Age, gender, education level and marital status


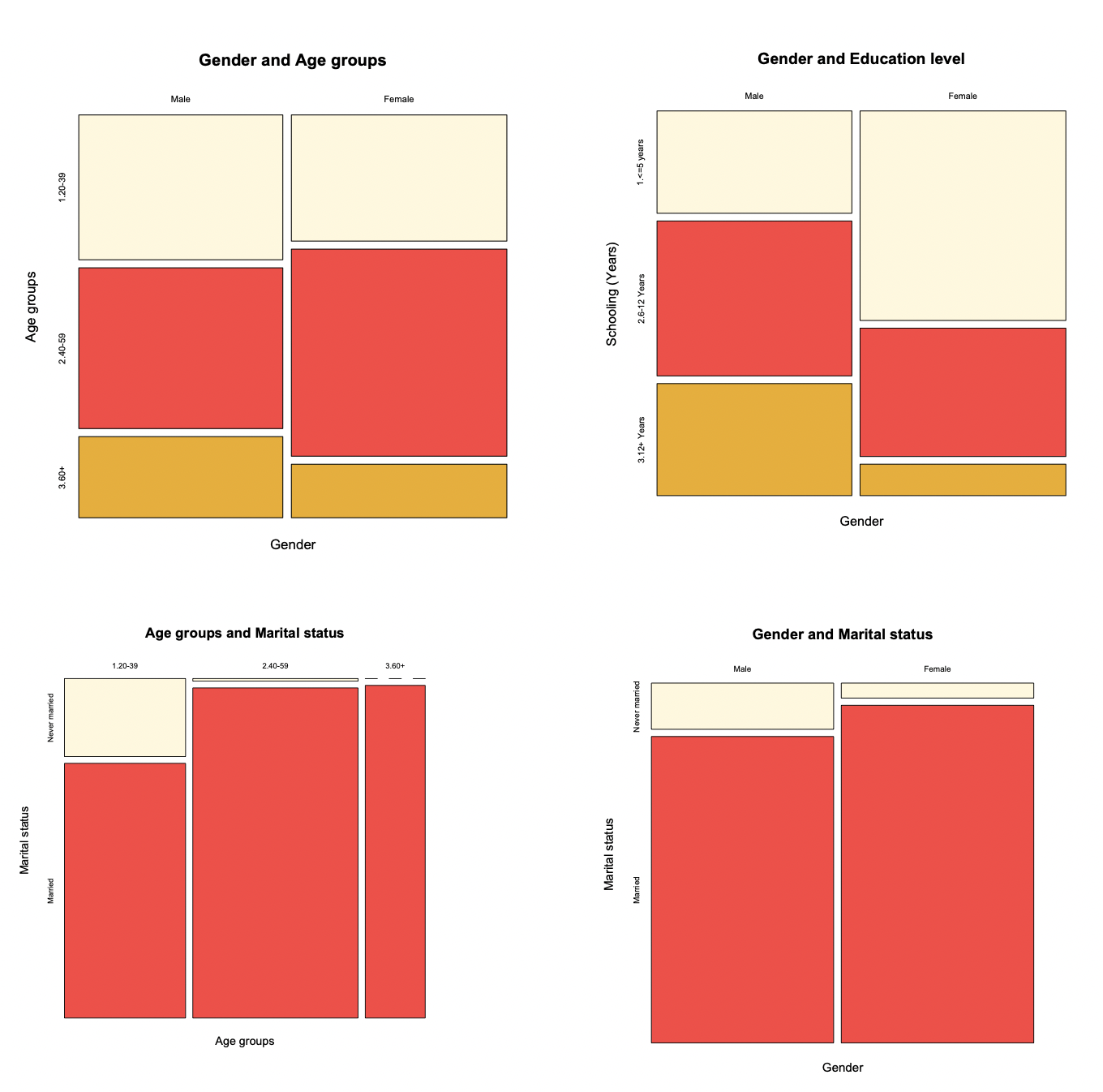

Supplement: S2 Fig — (DOCX) [file pone.0262221.s002.docx]

# S3: Multivariable logistic regression model with utilization of CAM exclusively


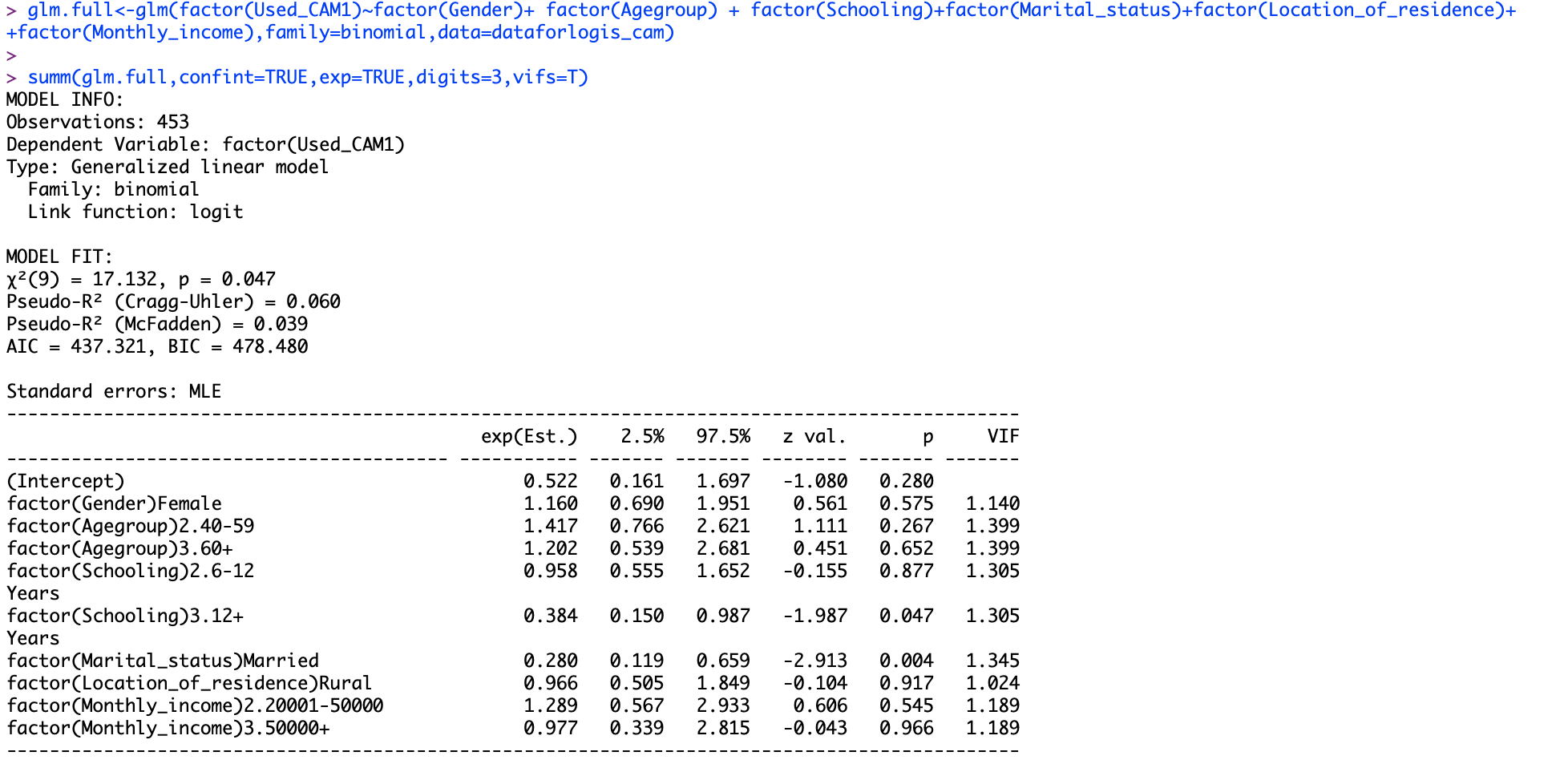

Supplement: S3 Fig — (DOCX) [file pone.0262221.s003.docx]
