# Supplementary material for: Prevalence and determinants of using complementary and alternative medicine for the treatment of chronic illnesses: A multicenter study in Bangladesh
Source: PLoS One. 2022 Jan 5;17(1):e0262221. doi: 10.1371/journal.pone.0262221 (PMC8730415; doi:10.1371/journal.pone.0262221)

# S4: Multivariable logistic regression model with utilization of both CM and CAM simultaneously


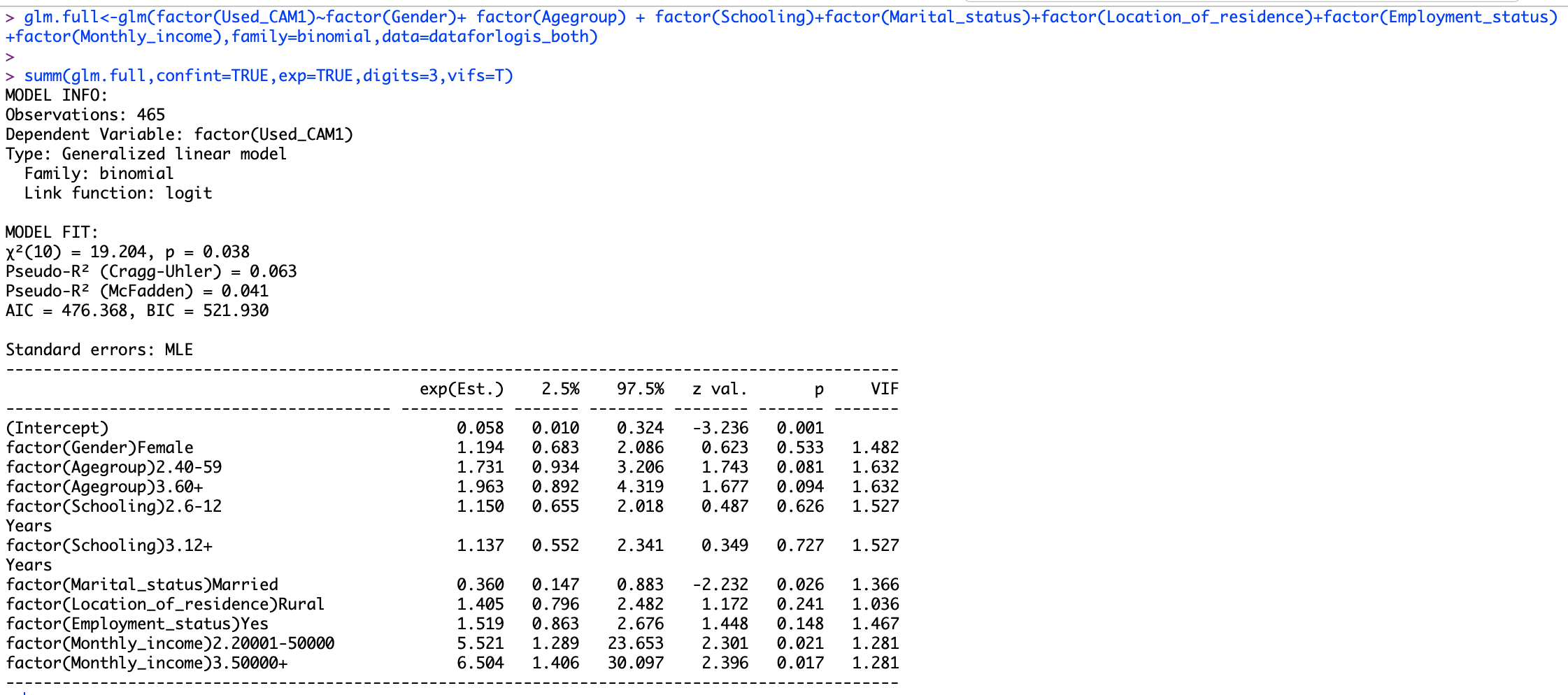

Supplement: S4 Fig — (DOCX) [file pone.0262221.s004.docx]
