# Supplementary material for: Prevalence and determinants of using complementary and alternative medicine for the treatment of chronic illnesses: A multicenter study in Bangladesh
Source: PLoS One. 2022 Jan 5;17(1):e0262221. doi: 10.1371/journal.pone.0262221 (PMC8730415; doi:10.1371/journal.pone.0262221)
Supplement: Attachment — Submitted filename: PONE-D-21-26170 - Abdelgadir.pdf [file pone.0262221.s005.pdf]

PLOS ONE

Prevalence and determinants of using complementary and alternative medicine for the treatment of chronic illness: A multicenter study in Bangladesh

--Manuscript Draft--

**Manuscript Number:**	PONE-D-21-26170
**Article Type:**	Research Article
**Full Title:**	Prevalence and determinants of using complementary and alternative medicine for the treatment of chronic illness: A multicenter study in Bangladesh
**Short Title:**	Use of complementary and alternative medicine for the treatment of chronic illness
**Corresponding Author:**	Ahmed Hossain, Ph.D.<br>North South University<br>Dhaka, N/A BANGLADESH
**Keywords:**	Conventional medicine; Complementary and alternative medicine; Noncommunicable diseases; Chronic illness; Bangladesh.
**Abstract:**	Background<br>While conventional medicine (CM) is commonly used to treat non-communicable diseases (NCDs), complementary and alternative medicine (CAM) is gaining popularity as a healthcare option in Bangladesh. We aimed to investigate the prevalence and factors associated with using CAM solely and using CAM in conjunction with CM for chronic illness treatment among NCD patients in Bangladesh.<br>Methods<br>A multicenter cross-sectional study was conducted, including 549 adults with a confirmed chronic illness diagnosis from three tertiary care hospitals in Dhaka city. Interviews were used to gather socio-demographic data, while medical records were used to get information on chronic illnesses. A multinomial logistic regression model was used to determine the associated factors of utilizing CAM primarily and CAM use in conjunction with CM to manage a chronic disease.<br>Results<br>Out of 549 NCD patients (282 women [51.4%], mean [standard deviation] age 45.4 [12.8] years), 180 (32.8%) ever used CAM for the treatment of chronic illness. Also, 15.3% of patients exclusively used CAM among the NCD patients, while 17.5% used CAM in conjunction with CM. Homeopathy medicine was the most prevalent type of treatment among CAM users (52.2%). Furthermore, 55.5% of CAM users said they used it because it had fewer side effects, and 41.6% trusted its effectiveness for chronic illness. Elderly patients (≥60 years) preferred CAM in conjunction with CM, but they did not rely only on CAM. According to the multinomial regression analysis, unmarried patients, predominantly in the younger age group, adopted CAM significantly for chronic illness treatment (RRR=0.29, 95% CI=0.12-0.71, reference= Unmarried). Patients in the high-income group used CAM in conjunction with CM (RRR=6.26, 95 percent CI=1.35 -18.90, reference: low-income), whereas patients in the high-income group did not rely on CAM alone (RRR=0.99, 95 percent CI=0.34-2.85).<br>Conclusion<br>Although CM remains the mainstream of health care in Bangladesh, CAM services play an essential role in people's health care, particularly in treating chronic illnesses. Physicians of Bangladesh should be aware that their patients may be using other services and be prepared to ask and answer questions regarding the risks and benefits of using CAM in addition to regular medical care. Thus, clinicians required to follow a best-practice guideline, which are currently not practiced in Bangladesh, when disseminating information regarding integrative techniques that combine CM and CAM approaches.
**Order of Authors:**	Md. Shahjalal
	Samar Kishor Chakma
	Tanvir Ahmed
	Irin Yasmin

Powered by Editorial Manager® and ProduXion Manager® from Aries Systems Corporation

<table>
<tr><td></td><td>Rashidul Alam Mahmud
Ahmed Hossain, Ph.D.</td></tr>
<tr><td>Additional Information:</td><td></td></tr>
<tr><td>Question</td><td>Response</td></tr>
<tr><td>Financial Disclosure

Enter a financial disclosure statement that describes the sources of funding for the work included in this submission. Review the submission guidelines for detailed requirements. View published research articles from PLOS ONE for specific examples.

This statement is required for submission and will appear in the published article if the submission is accepted. Please make sure it is accurate.

Unfunded studies
Enter: The author(s) received no specific funding for this work.

Funded studies
Enter a statement with the following details:
• Initials of the authors who received each award
• Grant numbers awarded to each author
• The full name of each funder
• URL of each funder website
• Did the sponsors or funders play any role in the study design, data collection and analysis, decision to publish, or preparation of the manuscript?
• NO - Include this sentence at the end of your statement: The funders had no role in study design, data collection and analysis, decision to publish, or preparation of the manuscript.
• YES - Specify the role(s) played.

* typeset</td><td>The author(s) received no specific funding for this work.</td></tr>
<tr><td>Competing Interests

Use the instructions below to enter a competing interest statement for this submission. On behalf of all authors, disclose any competing interests that could be perceived to bias this work—acknowledging all financial support and any other relevant financial or non-</td><td>The authors have declared that no competing interests exist.</td></tr>
</table>

financial competing interests.<br><br>This statement is required for submission and **will appear in the published article** if the submission is accepted. Please make sure it is accurate and that any funding sources listed in your Funding Information later in the submission form are also declared in your Financial Disclosure statement.<br><br>View published research articles from *PLOS ONE* for specific examples.<br><br>**NO authors have competing interests**<br><br>Enter: *The authors have declared that no competing interests exist.*<br><br>**Authors with competing interests**<br><br>Enter competing interest details beginning with this statement:<br><br>*I have read the journal's policy and the authors of this manuscript have the following competing interests: [insert competing interests here]*<br><br>* typeset	
**Ethics Statement**<br><br>Enter an ethics statement for this submission. This statement is required if the study involved:<br><br>• Human participants<br>• Human specimens or tissue<br>• Vertebrate animals or cephalopods<br>• Vertebrate embryos or tissues<br>• Field research<br><br>Write "N/A" if the submission does not require an ethics statement.<br><br>General guidance is provided below.<br>Consult the submission guidelines for	The participants gave verbal agreement for the study because they intended not to sign or leave fingerprints on any paper, and the many of them were analphabetic. The respondents were informed that all of the information obtained would be kept confidential and utilized solely for research purposes. They were, however, given a consent form that included specific contact information for the research investigators in case they had any questions in the future. The study protocol was approved by the Institutional Review Board of North South University in Bangladesh (2020/OR-NSU/IRB/1006).

detailed instructions. **Make sure that all information entered here is included in the Methods section of the manuscript.**<br><br>**Format for specific study types**<br><br>**Human Subject Research (involving human participants and/or tissue)**<br>• Give the name of the institutional review board or ethics committee that approved the study<br>• Include the approval number and/or a statement indicating approval of this research<br>• Indicate the form of consent obtained (written/oral) or the reason that consent was not obtained (e.g. the data were analyzed anonymously)<br><br>**Animal Research (involving vertebrate animals, embryos or tissues)**<br>• Provide the name of the Institutional Animal Care and Use Committee (IACUC) or other relevant ethics board that reviewed the study protocol, and indicate whether they approved this research or granted a formal waiver of ethical approval<br>• Include an approval number if one was obtained<br>• If the study involved *non-human primates*, add *additional details* about animal welfare and steps taken to ameliorate suffering<br>• If anesthesia, euthanasia, or any kind of animal sacrifice is part of the study, include briefly which substances and/or methods were applied<br><br>**Field Research**<br><br>Include the following details if this study involves the collection of plant, animal, or other materials from a natural setting:<br>• Field permit number<br>• Name of the institution or relevant body that granted permission	
**Data Availability**<br><br>Authors are required to make all data underlying the findings described fully available, without restriction, and from the time of publication. PLOS allows rare	Yes - all data are fully available without restriction

Powered by Editorial Manager® and ProduXion Manager® from Aries Systems Corporation

exceptions to address legal and ethical concerns. See the PLOS Data Policy and FAQ for detailed information.<br><br>A Data Availability Statement describing where the data can be found is required at submission. Your answers to this question constitute the Data Availability Statement and **will be published in the article**, if accepted.<br><br>**Important:** Stating 'data available on request from the author' is not sufficient. If your data are only available upon request, select 'No' for the first question and explain your exceptional situation in the text box.<br><br>Do the authors confirm that all data underlying the findings described in their manuscript are fully available without restriction?	
**Describe where the data may be found in full sentences. If you are copying our sample text, replace any instances of XXX with the appropriate details.**<br><br>• If the data are **held or will be held in a public repository**, include URLs, accession numbers or DOIs. If this information will only be available after acceptance, indicate this by ticking the box below. For example: *All XXX files are available from the XXX database (accession number(s) XXX, XXX.*).<br>• If the data are all contained **within the manuscript and/or Supporting Information files**, enter the following: *All relevant data are within the manuscript and its Supporting Information files.*<br>• If neither of these applies but you are able to provide **details of access elsewhere**, with or without limitations, please do so. For example:<br><br>*Data cannot be shared publicly because of [XXX]. Data are available from the XXX Institutional Data Access / Ethics Committee (contact via XXX) for researchers who meet the criteria for*	https://osf.io/x75jk/

access to confidential data.<br><br>*The data underlying the results presented in the study are available from (include the name of the third party and contact information or URL).*<br>• This text is appropriate if the data are owned by a third party and authors do not have permission to share the data.<br><br>* typeset	
Additional data availability information:	Tick here if the URLs/accession numbers/DOIs will be available only after acceptance of the manuscript for publication so that we can ensure their inclusion before publication.

Powered by Editorial Manager® and ProduXion Manager® from Aries Systems Corporation

Manuscript Click here to access/download;Manuscript;CAM Manuscript.docx

Original Research

Prevalence and determinants of using complementary and alternative medicine for the treatment of chronic illness: A multicenter study in Bangladesh

Short title: **Use of complementary and alternative medicine for the treatment of chronic illness**

Authors:

Md. Shahjalal[1,2], Samar Kishor Chakma[1,2], Tanvir Ahmed[1,2], Irin Yasmin[1,2], Rashidul Alam Mahumud[3,4], Ahmed Hossain[1,5]

[1]Department of Public Health, North South University, Dhaka-1229, Bangladesh

[2]Research Rats, Dhaka, Bangladesh

[3]NHMRC Clinical Trials Centre, Faculty of Medicine and Health, The University of Sydney, Camperdown, NSW-2006, Australia

[4]School of Business and Centre for Health Research, University of Southern Queensland, Toowoomba, QLD-4350, Australia

[5]Global Health Institute, North South University, Dhaka-1229, Bangladesh

Corresponding author:

Ahmed Hossain, PhD
Professor of Public Health &
Director, Global Health Institute
North South University, Dhaka-1229, Bangladesh.
Phone: +88 01729378236; Office: (8802) 5566-8200 Ext. 1936
Email: ahmed.hossain@utoronto.ca

Co-authors contact:

Md. Shahjalal: md.shahjalal3@northsouth.edu

Samar Kishor Chakma: samar.kishor@northsouth.edu

Tanvir Ahmed: tanvirahmedrpsc@y7mail.com

Irin Yasmin: irin.yasmin@gmail.com

Rashidul Alam Mahumud: rashed.mahumud@sydney.edu.au

Abstract

Background

While conventional medicine (CM) is commonly used to treat non-communicable diseases (NCDs), complementary and alternative medicine (CAM) is gaining popularity as a healthcare option in Bangladesh. We aimed to investigate the prevalence and factors associated with using CAM solely and using CAM in conjunction with CM for chronic illness treatment among NCD patients in Bangladesh.

Methods

A multicenter cross-sectional study was conducted, including 549 adults with a confirmed chronic illness diagnosis from three tertiary care hospitals in Dhaka city. Interviews were used to gather socio-demographic data, while medical records were used to get information on chronic illnesses. A multinomial logistic regression model was used to determine the associated factors of utilizing CAM primarily and CAM use in conjunction with CM to manage a chronic disease.

Results

Out of 549 NCD patients (282 women [51.4%], mean [standard deviation] age 45.4 [12.8] years), 180 (32.8%) ever used CAM for the treatment of chronic illness. Also, 15.3% of patients exclusively used CAM among the NCD patients, while 17.5% used CAM in conjunction with CM. Homeopathy medicine was the most prevalent type of treatment among CAM users (52.2%). Furthermore, 55.5% of CAM users said they used it because it had fewer side effects, and 41.6% trusted its effectiveness for chronic illness. Elderly patients (≥60 years) preferred CAM in conjunction with CM, but they did not rely only on CAM. According to the multinomial regression analysis, unmarried patients, predominantly in the younger age group, adopted CAM significantly for chronic illness treatment (RRR=0.29, 95% CI=0.12-0.71, reference= Unmarried). Patients in the high-income group used CAM in conjunction with CM (RRR=6.26, 95 percent CI=1.35 -18.90, reference: low-income), whereas patients in the high-income group did not rely on CAM alone (RRR=0.99, 95 percent CI=0.34- 2.85).

Conclusion

Although CM remains the mainstream of health care in Bangladesh, CAM services play an essential role in people's health care, particularly in treating chronic illnesses. Physicians of Bangladesh should be aware that their patients may be using other services and be prepared to ask and answer questions regarding the risks and benefits of using CAM in addition to regular medical care. Thus, clinicians required to follow a best-practice guideline, which are currently not practiced in Bangladesh, when disseminating information regarding integrative techniques that combine CM and CAM approaches.

Introduction

For millennia, complementary and alternative medicine (CAM) has been a popular way to meet people's basic healthcare needs [1]. CAM is the primary source of treatment for millions of individuals. In other cases, it is the only source of treatment due to a lack of adequate healthcare access, cultural differences, and healthcare costs [2]. Although conventional medicine (CM) has improved in recent decades, the use of CAM for illness prevention, control, and management has expanded around the world [2-3].

A considerable proportion of people in developed countries uses CAM healthcare services [4-6], which is equally true in developing countries [7-9]. Overall, the prevalence of using CAM ranged from 20% to 97.4% in South-East Asia [10]. More than 70% of the population in developing countries still depends on CAM treatment despite conventional medicine's progress [7]. According to a recent survey, 35.2% of diabetes patients in Bangladesh used CAM for their diabetic management [11].

Chronic diseases are caused by genetic, physiological, environmental, and behavioral variables, also known as non-communicable diseases (NCDs) [12]. NCDs place a considerable strain on the healthcare system in Southeast Asia, contributing to the high prevalence of early death (<70 years) [13]. Low- and middle-income countries (LMICs), such as Bangladesh, account for approximately 77% of overall NCD fatalities [12].

Moreover, NCDs are a significant source of morbidity and mortality in Bangladesh, accounting for 61% of all fatalities [14]. The most common NCDs include cardiovascular diseases, diabetes mellitus, cancer, and chronic respiratory diseases. As the proportions of these diseases increase year after year, these illnesses impose a significant burden on healthcare facilities [15]. Due to the increasing burden of NCDs, new challenges are arising around its long-term management [16]. Despite the enormous advancement of mainstream medicine, the usage of CAM continues to grow across the country [17]. Although CAM is not considered a part of mainstream health care systems, it has been employed in Bangladesh as an alternative to traditional treatment [17].

Patients seeking medical attention in Bangladesh are diverse, and disparity can be linked to physician bias or socioeconomic status and patients' understanding of risks and benefits, and healthcare system barriers [16]. Different CAM techniques, such as

Ayurvedic, Unani, Homeopathy, Naturopathy, and other folk practitioners, are commonly used in Bangladesh to address medical needs [17]. Bangladesh's geographical location and climate conditions are conducive to the growth and usage of CAM. Despite the widespread acceptance of CAM alongside traditional treatment, there is still a lack of data on the prevalence, utilization, and associated factors of using CAM for chronic illness among NCD patients in Bangladesh. Our study will look at the following goals: (i) Compute the prevalence of using CAM to manage chronic illness and categorizing patients by demographic subgroups, ii) Associated factors of exclusively CAM use for chronic disease, and iii) Factors associated with uses of CAM in conjunction with CM to manage chronic illness.

Methods and materials

Study design and settings

The study used a cross-sectional survey approach and was conducted among patients with NCDs who received healthcare services from the three tertiary care hospitals in Dhaka. The three hospitals were chosen conveniently, and the survey took place from January 12 to March 7, 2021. We set the following inclusion and exclusion criteria before data collection.

Inclusion criteria:

1. All of the participants had a chronic condition and had been taking medication for at least six months.
2. The chronic illnesses included: diabetes, hypertension, cardiovascular diseases (Coronary artery disease, Heart attack, Heart failure, Strokes or Rheumatic Heart Disease), chronic respiratory illness (Chronic obstructive pulmonary disease or asthma), chronic musculoskeletal disorders (chronic low back pain), rheumatic diseases, kidney diseases, and cancer.
3. The chronic condition of the participants was diagnosed using health care facilities such as hospitals, clinics, or CAM facilities, as well as by consultation with an expert clinical practitioner or CAM practitioner.
4. Age: 20-80 years.

5. The participants agreed to the consent form to respond to CAM uses and completed the interview.

Exclusion criteria:

1. We excluded pregnant mothers from the study.
2. Due to a lack of resources for immediate diagnosis of chronic or acute pain illness, individuals who reported pain for neuropathy or muscular pain were excluded.
3. Patients who reported self-medication for a chronic illness were excluded.
4. Patients with psychiatric disorders were ruled out by inquiring if they took any medicine for psychiatric symptoms or illegal drugs.

Sample size

The sample size for the survey was calculated using a 20% anticipated prevalence of CAM use for NCDs, with a 95% level of confidence and a 5% margin of error. The sample size was estimated using the epiR package of the R program 3.6.1. Bennett, Woods, and Smith described the formula for estimating sample size in a cluster sampling procedure (1991). The prevalence estimate is used to calculate the sample size required for the survey, resulting in the largest sample size possible. After adjustments, a total of at least 540 patients were included in the study. For the survey, we used a total of at least 200 patients per health facility after adjustments.

Sample selection

In the first stage, we chose three tertiary care hospitals from Dhaka city based on our conveniences. Islami Bank Hospital, Dhaka Medical College Hospital, and BIRDEM were the three hospitals. Patients were chosen in the second stage by systematic sampling, which field enumerators carried out. When patients arrived at the specified hospitals' NCD departments, they were assessed and asked about their diagnosis of chronic illness and duration of suffering for chronic disease. Then, using an alternative participant selection in the lines of visiting patients, at least 200 patients were selected from each hospital during the study period. At least five patients were interviewed each day during hospital working hours with the assistance of hospital physicians. A team of data collection enumerators interviewed the selected patients, and the hospital physician

confirmed the patient's NCD status. If the patient did not consent to data collection, they were not interviewed. Finally, data from 549 patients were gathered for analysis, and the flowchart of data collection is given in **Fig 1**.

[Fig 1: Flow chart of participants inclusion for analysis]

Measures

Independent variables

Independent variables included patients' sociodemographic characteristics and healthcare data on chronic illness. Gender, age group (20-39, 40-59, 60+ years), marital status (married, never married), years of schooling (1-5 years, 6-12 years, 12+ years of education), location of residence based on urbanicity (urban, rural), and employment status were among the sociodemographic characteristics collected in the study. Their monthly household income measured the respondents' economic situation. Monthly household income was divided into three categories: less than 20000 BDT (about US$ 238), 21000-50000 BDT, and >50000 BDT. We used two questions to determine the level of knowledge about CAM for chronic illness. The first is if the patients have heard of CAM for chronic illness treatment, and the second is whether they are aware of CAM's effectiveness in chronic illness management.

Outcome variable

The survey's outcome variable was the use of complementary and alternative medicine (CAM). We included the following mode of treatments in CAM definition: Ayurveda (Ayurvedic medicine), herbalism, homeopathy, Unani medicine, and traditional Chinese medicine. We did not include acupuncture, aromatherapy, massage therapy, meditation, and spiritual healing in our definition of CAM because they do not contain medication for treating chronic illness patients. We had three options for defining CAM: (i) Use CM exclusively for NCDs, (ii) Use CAM exclusively for NCDs, and (iii) Use CAM in conjunction with CM for NCDs. The outcome was determined using two questions. "Did you ever use complementary and alternative medicine (Ayurveda, herbalism, homeopathy, Unani medicine, and traditional Chinese medicine) for NCDs?" is the first question. The second question is, "Have you ever combined CAM and CM for NCDs?" The replies to each question were separated into two categories and recorded as yes or no.

Ethical permission

The participants gave verbal agreement for the study because they intended not to sign or leave fingerprints on any paper, and many of them were analphabetic. The respondents were informed that all of the information obtained would be kept confidential and utilized solely for research purposes. They were, however, given a consent form that included specific contact information for the research investigators in case they had any questions in the future. The study protocol was approved by the Institutional Review Board of North South University in Bangladesh (2020/OR-NSU/IRB/1006).

Statistical analysis

Data were analyzed using R 3.6.2. The questionnaire, R scripts, and data are available at https://osf.io/x75jk/. The CAM usage exclusively and CAM usage in conjunction with CM among the respondents were presented using descriptive statistics (frequencies, percentage). Therefore, we performed a cross-tabulation between the outcome measure (CM usage, CAM usage, and both CM and CAM usage) and the covariates. A multinomial regression model was fitted to examine the association of socio-demographic variables and CAM usage against chronic illness. The multinomial regression coefficient from the model was exponentiated and presented as relative risk ratios (RRR) along with corresponding 95% confidence intervals (CI). Here, RRR is defined as the ratio of the

probability of an outcome in the exposed group to the probability of an outcome in the unexposed group [18]. As some readers may find odds ratios (OR) easier to interpret than RRR, we included two binary logistic regressions examining, i) between CM usage exclusively and CAM usage exclusively, and ii) between CM usage exclusively and CAM usage in conjunction with CM in **Supplement S3 Table** and **S4 Table**, respectively. We also obtained variance inflation factors (VIF) in the logistic regression models to evaluate potential multicollinearity.

Results

Participants' characteristics

The mean ± SD age of the 549 participants was 45.4 ± 12.8 years. A total of 549 participants were interviewed, and their sociodemographic characteristics are given in **Table 1**. More than half of the participants (51.4%) were female. The majority of participants' age was between 40 years and 59 years (47.7%). Most had none or 1-5 years of schooling (42.6%). Besides, most of the participants were married (91.4%). The majority of participants were living in the urban area (81.4%). Most of the survey respondents were housewives or not working (65.6%), and their monthly household income was 20001-50000 BDT (71.9%). Moreover, patients' most common non-communicable diseases were: high blood pressure (35.2%) and diabetes (33.3%). In **Supplement S1**, the association between age, gender, education level, and marital status was discussed. The majority of the female participants appeared to have completed less than five years of schooling. Females had lower levels of education than males. In addition, the majority of the unmarried population was male and younger in age.

Prevalence of CAM use to manage chronic illness

Table 1 displays the prevalence of complementary and alternative medicine (CAM) use for chronic illness by sociodemographic categories. It appears that 180 of the 549 respondents (32.8%) utilized CAM for their chronic illness. Among the 180 CAM users, Homeopathy was utilized by 94 (52.2%), Ayurveda by 48 (26.6%), Unani by 29 (16%), and other CAM by 9 (5.2%) patients. **Supplement S2** displays a pie chart depicting the various types of CAM used by the patients. Furthermore, 84 (15.3 percent) of patients used CAM exclusively for sickness, whereas 94 (17.5 percent) utilized both CAM and CM

at the same time for the management of chronic conditions. The remaining 369 (67.2%) of the 549 patients relied solely on CM to manage their chronic illnesses.

According to the findings, 16.7% of 282 females and 13.9 percent of 267 males utilized CAM exclusively. Only CAM use was similar among persons in the 20-59 year age group, while it was low (13.7%) among the elderly (60+) age group. Both CM and CAM are used more frequently as people get older. Furthermore, among patients aged 20 to 39, the use of solely CM was common (68.8%).

Between respondents with 12+ years of schooling and those with less education, there was a notable change in the percentage of CAM use. Respondents with more than 12 years of schooling were less likely to use complementary and alternative medicine (6.7 percent). Respondents with a greater level of education, on the other hand, are more likely to use both CM and CAM to treat their sickness.

When compared to married respondents, a higher number of never-married respondents utilized CAM solely (27.7% for married versus 14.1% for never-married). Also, the percentage of never-married respondents who used both CAM and CM was greater. Furthermore, when compared to rural inhabitants, urban residents reported a higher percentage of CAM use (15.7% for urban versus 13.7% for rural). Rural residents, on the other hand, utilized a higher percentage of CAM usage in conjunction with CM than city dwellers (21.6% versus 16.6%).

Employed respondents used CAM at a lower rate than unemployed respondents. Only 10.1% of the 189 working respondents stated they used complementary and alternative medicine (CAM), compared to 18.1% of the 360 jobless respondents. However, employed respondents had a larger rate of both CM and CAM users than unemployed respondents (15.6%). Those with a monthly family income of more than 50000 BDT were more likely to employ both CM and CAM to treat their sickness. According to the findings, 108 (19.7%) of 549 patients did not know about CAM and had never utilized it to treat their ailment. On the other hand, 441 (80.3%) of the 549 patients knew about CAM, and around 40% of them had utilized it for their chronic illness.

[Table 1]

Distribution of CAM use by clinical features

Table 2 shows the distribution of CAM use and CM use for chronic illness according to the clinical characteristics of the patients. The majority of the participants visited hospitals with hypertension (35.2%) and diabetes problems (33.3%). Also, 78.8% of patients with hypertension were more likely to use CM alone, while 61.7% of diabetic patients were more likely to use CM alone. Diabetic individuals were also more likely to employ both CAM and CM to treat their ailment. Patients with respiratory sickness and musculoskeletal disorders were shown to be heavily reliant on CAM. These two categories of patients used CAM and CM for their illnesses at a higher rate than the other groups of diseases.

[Table 2]

Multinomial logistic regression model: Determinants of the utilization of CAM to manage NCDs

Table 3 shows the results of a multinomial logistic regression model for predicting the usage of CAM as well as the use of CAM in conjunction with CM. When compared to unmarried patients, married patients were 71% less likely to use exclusively complementary and alternative medicine (RRR=0.29, 95 percent CI=0.12-0.71). Similarly, married persons were 62% less likely to use both CAM and CM than unmarried people (RRR=0.38, 95 percent CI=0.16 -0.90). Furthermore, patients with a monthly household income of more than 50000 BDT were 6.26 times more likely than low-income families to utilize both CAM and CM (RRR=6.26, 95 percent CI=1.35 -28.90). Patients' gender, age, education, locality, and employability, on the other hand, were unrelated to their use of CAM.

Reasons for CAM use to manage chronic illness

Patients who had previously used complementary and alternative medicine for their disease were questioned about their motives for doing so, and the results are given in Figure 2. For such a question, multiple responses were collected. According to the findings, 55.5% of the 180 CAM users said they used it to avoid adverse side effects. Furthermore, 41.6% of the CAM users stated it was effective in the management of

chronic illnesses, while 38.8% said it was easier to obtain from local services and less expensive.

[Figure 2: Reasons for CAM use among patients]

Discussion

This study investigates the prevalence and pattern of complementary and alternative medicine (CAM) usage and the factors that influence CAM use among Bangladeshi adult patients with NCDs. In the study, one-third of the participants had utilized CAM to manage chronic diseases at some point in their lives. This result is similar to what was previously reported among diabetic patients in Bangladesh (35.2%) [11]. A study in Malaysia found that 63.9% of patients with chronic conditions used CAM, and another study in North-India found the rate was 53.2%, which is significantly higher than our estimate [19-20]. Besides, Singapore (22.7%) and South Africa (27.2%) had lower rates of CAM use among patients with chronic illness [21-22]. Moreover, the most prevalent reasons for CAM use were a few side effects, improved efficacy, and that CAM is less expensive and more readily available. These causes are comparable to the findings of earlier investigations [11, 17-21].

According to [illegible] study, patients with NCDs exhibited a significant intention to use CAM in conjunction with CM (17.5%) to manage their chronic diseases. A survey in the United States indicated that 54.9% of individuals used CAM in combination with CM [23], while another study in North-India found that 49.8% of patients used CAM in conjunction with CM [20]. This finding raises concerns regarding drug interactions between these two types of treatment methods. To establish the efficacy and safety of combined treatment, it appears necessary to look into the scientific data.

According to the findings of this survey, socio-demographic characteristics such as marital status and a high monthly household income were significantly related to CAM use in conjunction with CM among NCD patients, which is similar to earlier studies from Malaysia, Nepal, and Pakistan [19, 24-25]. These findings could point to a trend among CAM users for high-income people to seek every available alternative for their health care benefit and well-being. However, a recent study found that, in addition to the four income

levels, both the lowest and highest socioeconomic groups in China demonstrated an increased preference for CAM [26].

The majority of females used CAM solely when they were younger, while most males preferred to use CAM in combination with CM. A study in the United States found that females were more likely to use CAM than males [23]. On the other hand, a survey conducted in North India and another study conducted in Bangladesh with diabetic patients observed no gender differences in CAM use [11, 20].

The global epidemic of chronic diseases is driven by population aging, yet there is significant untapped potential to change the relationship between chronological age and health [27]. As a result, obtaining medical attention at the onset of sickness is critical for managing a chronic condition. Our results suggest the percentage of CAM use in combination with CM was high among the older (≥60) age group patients. This result is consistent with the other studies in South-East Asian countries like India, Malaysia, and China [22, 19, 26]. One possible explanation is that younger people seek alternative and low-cost remedies for mild issues, while elderly patients seek traditional medical aid for severe complications.

According to the data, patients with a high level of education were more likely to use combination CAM and CM. Patients with an insufficient level of education, on the other hand, were more likely to use CAM solely. This finding backs with a previous study conducted in Bangladesh, South Africa, and India, which found that primary school students were more likely to use CAM than those with a higher degree [11,20-21]. Another study in Canada saw an increased level of education increases the use of CAM in conjunction with CM [28]. Lack of knowledge was substantially linked to lower education and income levels as a factor for non-use of CAM.

Strengths and limitations

Our research has several positive aspects. First, we gathered data with the cooperation of university graduate enumerators and physicians from the surveyed hospitals. By involving physicians from hospitals, we were able to reduce information bias. Second, we used a systematic sampling strategy to find patients within a hospital. Third, we asked if CAM was used in combination with CM to determine CAM usage, which helped us

categorize CAM users in three groups, which was missing in most literature. Several limitations should be taken into account when interpreting the results. First, our study could not capture the trend of CAM usage over time, as we considered a cross-sectional study. Second, we did not view the availability of CAM services. This could be significant because the prevalence of CAM usage among respondents may be mitigated by accessing or using available health services. Also, we did not consider other variables such as duration of chronic diseases, family history of treatment methods, access to health services, etc. These variables could have confounding effects on the prevalence of using CAM or using CAM in combination with CM.

Conclusion

Although patients with chronic diseases utilized a high percentage of CM, CAM is also widely used for chronic illness treatment in Bangladesh. A large proportion of patients viewed the use of CAM in conjunction with CM as complementary rather than alternative. As a result, physicians in Bangladesh should be aware that their patients may be utilizing other services and be ready to ask and answer questions about the risks and advantages of using CAM in addition to traditional medical care. We emphasize clinicians' requirement to follow a best-practice guideline when disseminating information regarding integrative techniques that combine CM and CAM approaches to manage chronic illness.

Supporting information

Figure S1: Types of CAM utilized by the patients **(Supplement.docx)**

Appendix S1: Relationship between age, gender, education level and marital status **(Supplement.docx)**

Appendix S2: Multivariable logistic regression model with utilization of CAM exclusively **(Supplement.docx)**

Figure S1: Multivariable logistic regression model with utilization of CAM in combination with CM **(Supplement.docx)**

Acknowledgments

All authors acknowledge the graduate students from North South University who were involved in data collection. We are also thankful to the hospital authorities for their approval and support during data collection. We would also like to thank the … for insightful comments that improved the presentation and clarity of our manuscript.

References

1. Shorofi SA, Arbon P. Complementary and alternative medicine (CAM) among Australian hospital-based nurses: knowledge, attitude, personal and professional use, reasons for use, CAM referrals, and socio-demographic predictors of CAM users. *Complement Ther Clin Pract*. 2017 May;27:37-45. doi: 10.1016/j.ctcp.2017.03.001. Epub 2017 Mar 30. PMID: 28438278.

2. WHO global report on traditional and complementary medicine 2019. Geneva: World

Health Organization; 2019. Licence: CC BY-NC-SA 3.0 IGO. Available at: https://www.who.int/traditional-complementary-integrative-medicine/WhoGlobalReportOnTraditionalAndComplementaryMedicine2019.pdf (accessed July 03, 2021)

3. Chung VCH, Wong CHL, Zhong CCW, Tjioe YY, Leung TH, Griffiths SM. Traditional and complementary medicine for promoting healthy ageing in WHO Western Pacific Region: Policy implications from utilization patterns and current evidence. *Integr Med Res*. 2021 Mar;10(1):100469. doi: 10.1016/j.imr.2020.100469. Epub 2020 Jul 4. PMID: 32874912; PMCID: PMC7452244.

4. Ernst E, White A. The BBC survey of complementary medicine use in the UK. *Complement Ther Med*. 2000 Mar;8(1):32-6. PMID: 10812758.

5. Eisenberg DM, Davis RB, Ettner SL, et al. Trends in Alternative Medicine Use in the United States, 1990-1997: Results of a Follow-up National Survey. *JAMA*. 1998;280(18):1569–1575. doi:10.1001/jama.280.18.1569

6. Yamashita H, Tsukayama H, Sugishita C. Popularity of complementary and alternative medicine in Japan: a telephone survey. *Complement Ther Med*. 2002 Jun;10(2):84-93. doi: 10.1054/ctim.2002.0519. PMID: 12481956.

7.Bodeker G, Kronenberg F. A public health agenda for traditional, complementary, and alternative medicine. *Am J Public Health*. 2002 Oct;92(10):1582-91. doi: 10.2105/ajph.92.10.1582. PMID: 12356597; PMCID: PMC3221447.

8. Chen, FP., Chen, TJ., Kung, YY. et al. Use frequency of traditional Chinese medicine in Taiwan. *BMC Health Serv Res* 7, 26 (2007). https://doi.org/10.1186/1472-6963-7-26

9. Shaikh BT, Hatcher J. Complementary and Alternative Medicine in Pakistan: Prospects and Limitations. *Evid Based Complement Alternat Med.* 2005 Jun;2(2):139-142. doi: 10.1093/ecam/neh088. Epub 2005 Apr 20. PMID: 15937553; PMCID: PMC1142200.

10. Karl Peltzer & Supa Pengpid (2015) Utilization and Practice of Traditional/Complementary/Alternative Medicine (T/CAM) in Southeast Asian Nations (ASEAN) Member States, *Studies on Ethno-Medicine*, 9:2, 209-218, DOI: 10.1080/09735070.2015.11905437

11. Rafi, M.A., Azad, D.T., Bhattacharjee, M. et al. A hospital-based study on complementary and alternative medicine use among diabetes patients in Rajshahi, Bangladesh. *BMC Complement Med Ther* 20, 219 (2020). https://doi.org/10.1186/s12906-020-03021-3

12. WHO Fact sheets, 2021, Noncommunicable diseases, Available at https://www.who.int/news-room/fact-sheets/detail/noncommunicable-diseases (Accessed July 03, 2021)

13. World Health Organization. Noncommunicable Diseases (NCD). (2019). Available online at: https://www.who.int/gho/ncd/mortality_morbidity/en/ (Accessed July 03, 2021).

14. Alam D, Robinson H, Kanungo A, Hossain MD, Hassan M. Health Systems Preparedness for responding to the growing burden of non-communicable disease-a case study of Bangladesh. Melbourne: Place? : Nossal Institute for Global Health; 2013. p.1–25. Available at: http://ni.unimelb.edu.au/__data/assets/pdf_file/0008/720656/WP25.pdf. (Accessed July 03, 2021)

15. Samira Humaira Habib, Soma Saha, Burden of non-communicable disease: Global overview, *Diabetes & Metabolic Syndrome: Clinical Research & Reviews*, Volume 4, Issue 1, 2010, Pages 41-47, ISSN 1871-4021, https://doi.org/10.1016/j.dsx.2008.04.005.

16. Althoff KN, Smit M, Reiss P, Justice AC. HIV and ageing: improving quantity and quality of life. *Curr Opin HIV AIDS.* 2016 Sep;11(5):527-536. doi: 10.1097/COH.0000000000000305. PMID: 27367780; PMCID: PMC5084838.

17. Haque MI, Chowdhury ABMA, Shahjahan M, Harun MGD. Traditional healing practices in rural Bangladesh: a qualitative investigation. *BMC Complement Altern Med.* 2018;18(1):62. Published 2018 Feb 15. doi:10.1186/s12906-018-2129-5.

18. Camey SA, Torman VB, Hirakata VN, Cortes RX, Vigo A. Bias of using odds ratio estimates in multinomial logistic regressions to estimate relative risk or prevalence ratio and alternatives. *Cad Saude Publica.* 2014 Jan;30(1):21-9. doi: 10.1590/0102-311x00077313. PMID: 24627010.

19. Hasan SS, Ahmed SI, Bukhari NI, Loon WC. Use of complementary and alternative medicine among patients with chronic diseases at outpatient clinics. *Complement Ther*

Clin Pract. 2009 Aug;15(3):152-7. doi: 10.1016/j.ctcp.2009.02.003. Epub 2009 Mar 3. PMID: 19595416.

20. Nailwal D, B VR, Gupta A. Patterns and predictors of complementary and alternative medicine use in people presenting with the non-communicable disease in an urban health facility, North India. *J Public Health Res*. 2021 Mar 2;10(1):2109. doi: 10.4081/jphr.2021.2109. PMID: 33708751; PMCID: PMC7941049.

21. GBW Lee, TC Charn, ZH Chew, TP Ng, Complementary and alternative medicine use in patients with chronic diseases in primary care is associated with perceived quality of care and cultural beliefs, *Family Practice*, Volume 21, Issue 6, December 2004, Pages 654–660, https://doi.org/10.1093/fampra/cmh613

22. Gail D. Hughes, Oluwaseyi M. Aboyade, Roxanne Beauclair, Oluchi N. Mbamalu, Thandi R. Puoane, Characterizing Herbal Medicine Use for Noncommunicable Diseases in Urban South Africa, *Evidence-Based Complementary and Alternative Medicine*, vol. 2015, Article ID 736074, 10 pages, 2015. https://doi.org/10.1155/2015/736074

23. Barnes PM, Powell-Griner E, McFann K, Nahin RL. Complementary and alternative medicine use among adults: United States, 2002. Adv Data. 2004 May 27;(343):1-19. PMID: 15188733.

24. Kadayat, T.M., Bist, G., Parajuli, A. et al. Patterns and perception of complementary and alternative medicine use by patients in western Nepal. *J Public Health* 20, 297–303 (2012). https://doi.org/10.1007/s10389-011-0446-y

25. Shaikh BT, Hatcher J. Complementary and Alternative Medicine in Pakistan: Prospects and Limitations. *Evid Based Complement Alternat Med*. 2005 Jun;2(2):139-142. doi: 10.1093/ecam/neh088. Epub 2005 Apr 20. PMID: 15937553; PMCID: PMC1142200.

26. Xin, B., Mu, S., Tan, T. et al. Belief in and use of traditional Chinese medicine in Shanghai older adults: a cross-sectional study. *BMC Complement Med Ther* 20, 128 (2020). https://doi.org/10.1186/s12906-020-02910-x

27. Prince MJ, Wu F, Guo Y, Gutierrez Robledo LM, O'Donnell M, Sullivan R, Yusuf S. The burden of disease in older people and implications for health policy and practice. *Lancet*. 2015 Feb 7;385(9967):549-62. doi: 10.1016/S0140-6736(14)61347-7. Epub 2014 Nov 6. PMID: 25468153.

28. Metcalfe, A., Williams, J., McChesney, J. et al. Use of complementary and alternative medicine by those with a chronic disease and the general population - results of a national population based survey. BMC Complement Altern Med 10, 58 (2010). https://doi.org/10.1186/1472-6882-10-58

Table 1. Distribution of participants according to CAM and CM users

Participants' characteristics	CM Only (n=369, 67.2%)	CAM Only (n=84, 15.3%)	CAM in conjunction with CM (n=96, 17.5%)	Total (n=549)
**Gender**				
Male	179 (67.0%)	37 (13.9%)	51 (19.1%)	267 (48.6%)
Female	190 (67.4%)	47 (16.7%)	45 (16.0%)	282 (51.4%)
**Age (years)**				
20-39	132 (68.8%)	30 (15.6%)	30 (15.6%)	192 (35.0%)
40-59	173 (66.0%)	41 (15.6%)	48 (18.3%)	262 (47.7%)
≥60	64 (67.4%)	13 (13.7%)	18 (18.9%)	95 (17.3%)
**Schooling**				
<=5 years	158 (67.5%)	41 (17.5%)	35 (15.0%)	234 (42.6%)
6-12 years	137 (65.2%)	36 (17.1%)	37 (17.6%)	210 (38.3%)
12+ years	74 (70.5%)	7 (6.7%)	24 (22.9%)	105 (19.1%)
**Marital status**				
Married	346 (68.9%)	71 (14.1%)	85 (16.9%)	502 (91.4%)
Never married	23 (48.9%)	13 (27.7%)	11 (23.4%)	47 (8.6%)
**Location of residence**				
Urban	303 (67.8%)	70 (15.7%)	74 (16.6%)	447 (81.4%)
Rural	66 (64.7%)	14 (13.7%)	22 (21.6%)	102 (18.6%)
**Employment status**				
No employment	239 (66.4%)	65 (18.1%)	56 (15.6%)	360 (65.6%)
Had an employment	130 (68.8%)	19 (10.1%)	40 (21.2%)	189 (34.4%)
**Monthly household income (BDT)**				
≤20000	40 (80.0%)	8 (16.0%)	2 (4.0%)	50 (9.1%)
20001-50000	259 (65.6%)	66 (16.7%)	70 (17.7%)	395 (71.9%)
50000+	70 (67.3%)	10 (9.6%)	24 (23.1%)	104 (18.9%)
**Knowledge on CAM**				
No	108 (96.3%)	0 (0%)	4 (3.7%)	108 (19.7%)
Yes	265 (65.6%)	84 (19.0%)	92 (20.9%)	441 (80.3%)

Table 2: The percentage of the use of CAM by clinical features of the patients.

**Clinical features**	**CM Only (n=369, 67.2%)**	**CAM Only (n=84, 15.3%)**	**CAM in conjunction with CM (n=96, 17.5%)**	**Total (n=549)**
High blood pressure/ Hypertension	152 (78.8%)	33 (17.1%)	8 (4.1%)	193 (35.2%)
Diabetes	113 (61.7%)	21 (11.5%)	49 (26.8%)	183 (33.3%)
CVD	21 (65.6%)	8 (25.0%)	3 (9.4%)	32(5.8%)
Respiratory illness	9 (40.9%)	6 (27.3%)	7 (31.8%)	22 (4%)
Musculoskeletal disorder	13 (38.2%)	7 (20.6%)	14 (41.2%)	34 (6.2%)
Others (e.g., Cancer, CKD, Rheumatic diseases)	61 (71.8%)	9 (10.6%)	15 (17.6%)	85 (15.5%)

Table 3. Multinomial logistic regression model to understand associated factors on the use of CAM

**Participants' characteristics**	**CAM only**		**CAM in conjunction with CM**	
	**RRR**	**95% CI**	**RRR**	**95% CI**
**Gender (reference: female)**				
Male	0.96	0.54 - 1.72	1.17	0.68 -2.02
**Age group (reference: 20-39 years)**				
40-59 years	1.34	0.72- 2.50	1.66	0.91-3.01
≥60 years	1.02	0.44- 2.37	1.93	0.88 -4.20
**Marital status (reference: never married)**				
**Married**	**0.29**	**0.12- 0.71**	**0.38**	**0.16 -0.90**
**Schooling (reference: <=5 years)**				
6-12 years	0.96	0.56- 1.67	1.15	0.66 -2.01
12+ years	0.40	0.15- 1.05	1.21	0.71 -2.29
**Location of residence (reference: urban)**				
Rural	0.99	0.52- 1.90	1.36	0.77 -2.40
**Employment status (reference: Housewife or not employed)**				
Employed	0.61	0.32- 1.17	1.50	0.86 -2.62
**Monthly family income (reference: <=20000 BDT)**				
20001-50000 BDT	1.30	0.57- 2.96	**5.39**	**1.26 -13.06**
50000+ BDT	0.99	0.34- 2.85	**6.26**	**1.35 -18.90**

Figure

Click here to access/download;Figure;flowchart.pdf

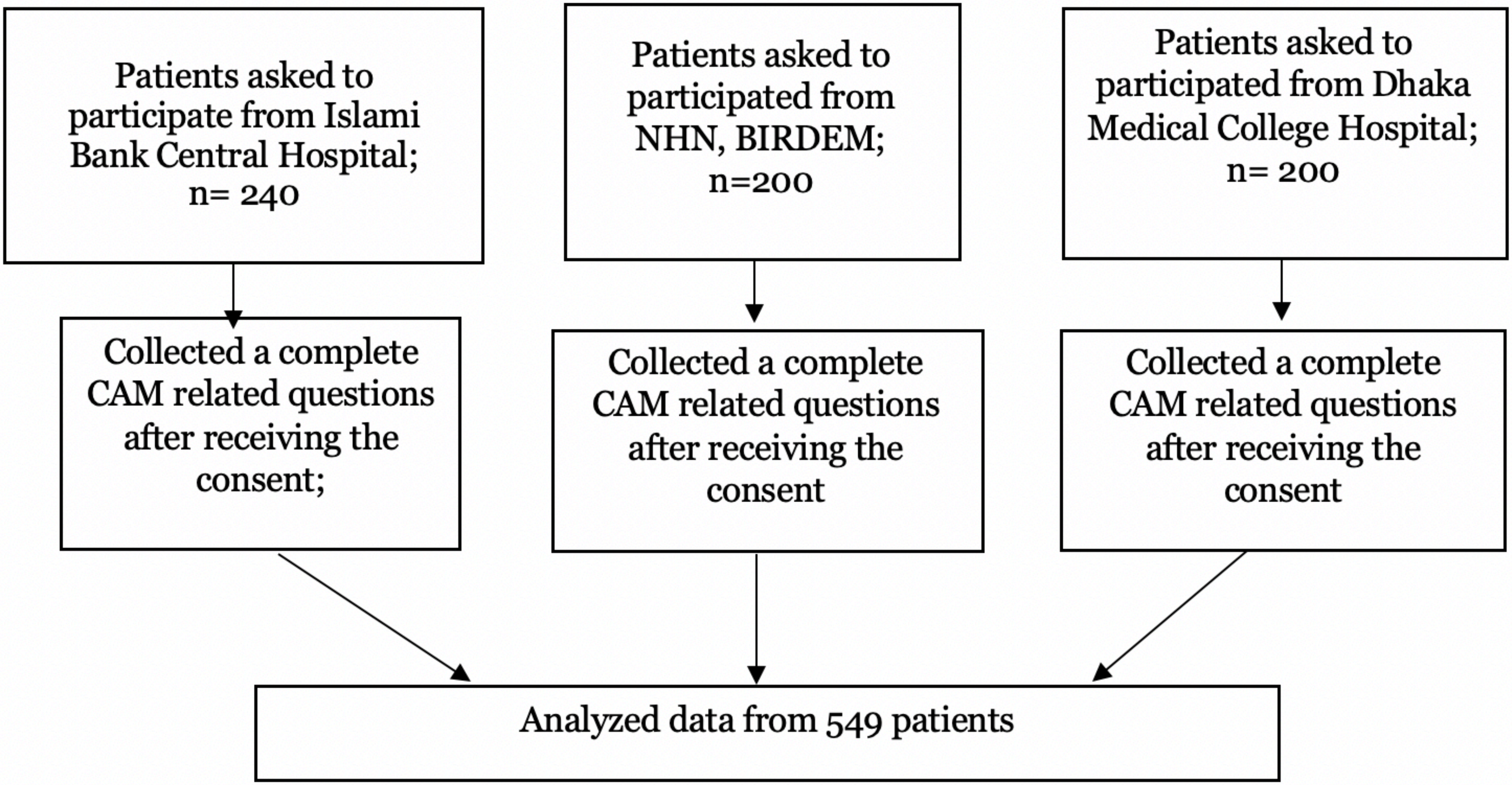

Figure

Click here to access/download;Figure;Reasons.pdf

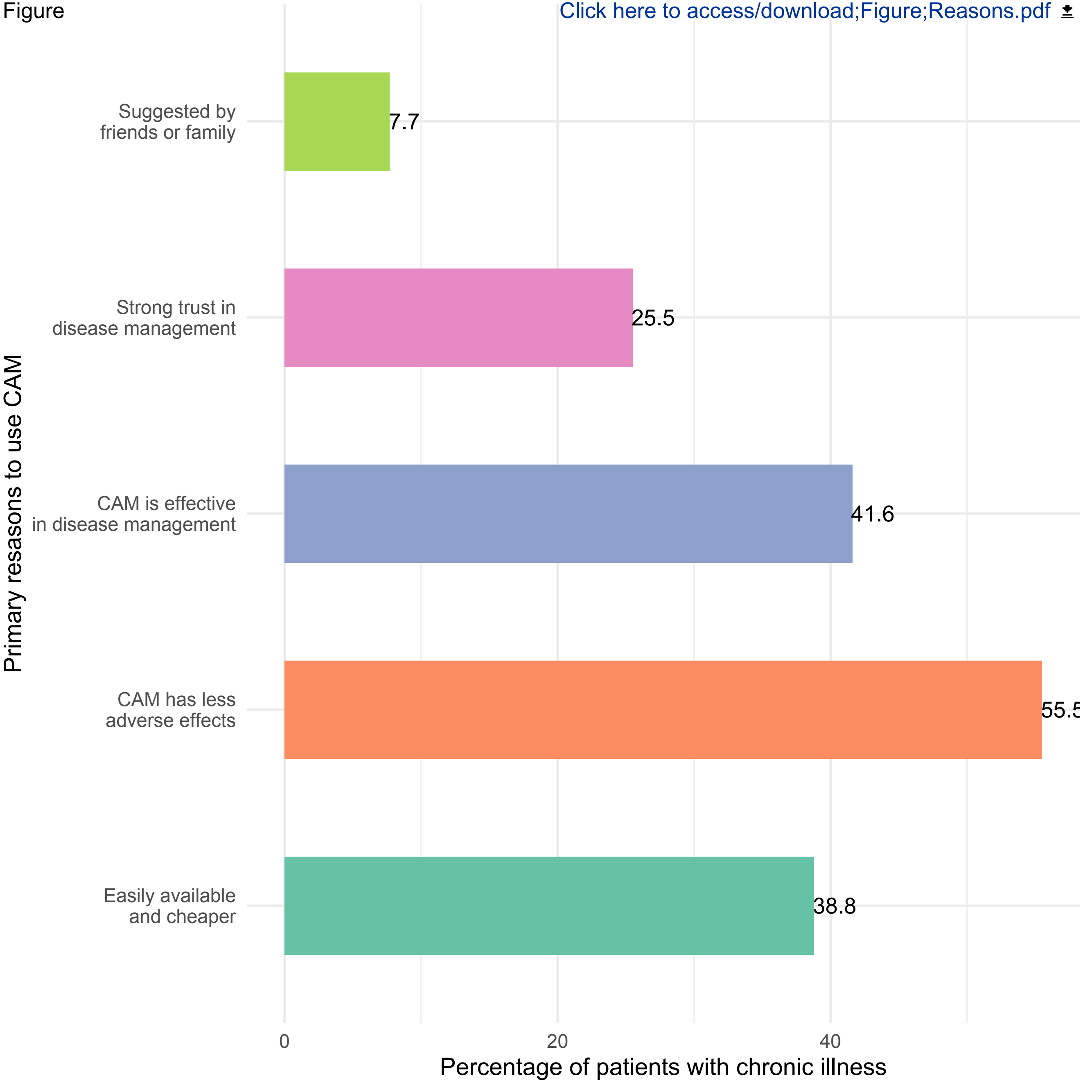

Click here to access/download
Supporting Information
Supplement.docx
