# Supplementary material for: Prevalence and determinants of using complementary and alternative medicine for the treatment of chronic illnesses: A multicenter study in Bangladesh
Source: PLoS One. 2022 Jan 5;17(1):e0262221. doi: 10.1371/journal.pone.0262221 (PMC8730415; doi:10.1371/journal.pone.0262221)
Supplement: Attachment — Submitted filename: Reviewer report 1.docx [file pone.0262221.s006.docx]

December 18, 2021

Bashir Alsiddig Yousef, Ph.D

Academic Editor

PLOS ONE

**PONE-D-21-26170**

**Prevalence and determinants of using complementary and alternative**

**medicine for the treatment of chronic illnesses: A multicenter study in Bangladesh**

Dear Professor Bashir Alsiddig Yousef,

Thank you very much for your editorial suggestions and the reviewers’ comments. They were accommodating. Please find enclosed an itemized list of responses along with the revised manuscript.

In our response to the reviewer, we used regular font for the comments/questions by the referees and regular, bold font for our responses, which are shown immediately following the questions/comments.

Thank you once again for the opportunity to submit a revised manuscript.

Ahmed Hossain, PhD

Professor, Department of Public Health

Director, Global Health Institute.

North South University.

# Reviewer #1:

 - In introduction, add a concise definition for CAM

**Authors: Thank you very much for your insightful comment. We included the following definition in the introduction:**

***Complementary and alternative medicines (CAMs) are a non-mainstream approach that do not fit under the umbrella of traditional medicine. Acupuncture, homeopathy, aromatherapy, meditation, and colonic irrigation are examples of these medications and treatments.***

-In the sampling (page 5), hospital selection based on convivence, which may decrease the external validity in the study.

**Authors: Thank you very much for your wonderful comment. We agree with you that the convenient sampling has its own limitations of taking under- or over-representation sample of the population. It is the most commonly used sampling technique in public health as it is incredibly prompt, uncomplicated, and economical. To reduce the biasness in our study we choose three popular public hospitals in Dhaka. We also reduced bias in sampling by using systematic sampling in patients’ selection. Thus, we used both convenience sampling and probability sampling techniques to draw a more accurate result. The probability aspect used, along with convenience sampling, helped us to reduce bias in the results.**

- In methodology, Study pupation, (page 5) pregnant excluded, Why?

**Authors: We excluded pregnant women from the study to reduce scientific complexity, as there is a chance that a patient seeking medical attention for pregnancy rather than chronic illness would be included. Pregnant women are scientifically complex, owing to their physiologic and ethical complexity. Also, pregnant women with substantial medical issues (e.g., diabetes, inflammatory bowel disease, depression, and epilepsy) may have a false positive reaction as a chronic illness patient. Because these factors could have a negative impact on the study, pregnant women were excluded in our study.**

Aslo individuals using self medications were excluded, why?.
**Authors: Patients who self-medicate do not engage in health-care consultations, which may obstruct our goal of determining the way to seek medical attention. Patients who self-medicate were excluded from the study to avoid the complexities of finding practice of medical care for a chronic illness.**

# Reviewer #2:

Attached comments for the author:
Minor change in the tiltle " illnesses".

**Authors: Many thanks. Changed it to illnesses.**
Minor comments in abstract.
Results : should be explain with more clear figure.

**Authors: There were two graphs shown in the manuscript and two more figures were presented in the supplement. The flow chart of sample selection is shown in the first figure, and the Barplot of reasons for CAM use is shown in the second figure. In the manuscript, both figures were explained and clearly presented. Besides, the supplementary figures were explained in the manuscript.**

The type of referencing should be consistent.

**Authors: The references are verified with Pubmed suggested AMA style.**
